# IL-Iβ+3954 C/T Polymorphism and Its Clinical Associations in Egyptian Sickle Cell Disease Patients

**Published:** 2019-01-01

**Authors:** Rasha Abdel-Raouf Abdel-Aziz Afifi, Yasser Mohamad Sedky, Hesham Abd-ELKareem, Shahira Kamal Anis Botros

**Affiliations:** 1 Department of Pediatrics, Cairo University, Cairo, Egypt; 2 Faculty of Medicine, Cairo University, Cairo, Egypt; 3 Department of Clinical Pathology, Cairo University, Cairo, Egypt

**Keywords:** Sickle cell disease, Interleukin-1 beta, Single nucleotide polymorphism, Hypertension, Pulmonary

## Abstract

**Background:** Sickle cell disease (SCD) is a hereditary disorder characterized by hemolytic anemia with different clinical manifestations. Patients with SCD exhibit a chronic inflammatory state and reduced length and quality of life**. **Interleukin-1 β (IL-1β) is important in acute and chronic diseases; and its single nucleotide polymorphisms (SNP) have been considered as predictors of prognosis in several inflammatory conditions. This study aimed at exploring IL-1β (+3954C/T) SNP as a potential genetic modifier and/or predictor of SCD clinical and laboratory phenotypes.

**Materials and Methods: **This cross-sectional study involved 50 SCD patients and 50 age, sex and ethnicity-matched healthy individuals. IL-1β (+3954C/T) SNP was identified by PCR-RFLP. Associations between IL-1β (+3954 C/T) SNP and the clinical and laboratory profiles of patients with SCD were studied.

**Results: **It was found that the homozygous mutant genotype TT was significantly higher in cases compared to controls [13(26%) vs. 3(6%) respectively; p=0.006, OR (95%CI): 5.505(1.460-20.756)]. The homozygous mutant genotype TT was associated with a higher mean pulmonary arterial pressure when compared to the CC and CT genotype (42.62 vs. 33.49 mmHg, p<0.001).

**Conclusion: **There is an increased prevalence of the mutant genotype of IL-1β +3954 SNP in Egyptian SCD patients. Regarding disease complications, the mutant genotype was more prevalent in cases complicated by pulmonary hypertension. These findings point to the possible role of IL-1β +3954 SNP in the pathophysiology of SCD and its manifestations.

## Introduction

 Sickle cell disease (SCD) is a generic term used to define a group of genetic changes characterized by the dominance of hemoglobin S (Hb S). Hb S is characterized by a missense mutation in position 6 of the β chain, in which the amino acid glutamic acid is replaced by valine^1^. 

SCD is a hereditary disorder characterized by hemolytic anemia with different clinical manifestations; and is considered as a chronic inflammatory disease. Patients with SCD exhibit a chronic inflammatory state and reduced length and quality of life^2^. SCD is one of the most important single gene disorders of human beings. It is estimated that 75–85% of children born with SCD are born in Africa^3^. The highest prevalence of sickle-cell trait (SCT) in Africa occurs between the latitudes of 15° North and 20° South, where the prevalence ranges from 10% to 40% of the population^4^. In Egypt, The prevalence of SCD is 0.3%. HbS carrier rates vary from 9 to 22 % in some regions^5^. In 1951, Abbasy reported the first case of SCD in Egypt^6^. SCD is characterized by hemolysis, higher risk of infections and recurrent vaso-occlusive crisis (VOC) with pain, which result in chronic organ damage. Several studies have identified factors associated with this heterogeneity in order to predict the clinical outcome and/or prognosis of the patients^7^. 

The hallmark abnormality of SCD is the polymerization of deoxygenated hemoglobin S and aggregation into fibers. This causes drastic change in hemoglobin solubility that leads to heterogeneities in cell shape and density. Despite being a monogenic disease, patients with SCD have a substantial phenotypic variability^8^. 

Interleukin-1 β (IL-1 β) is a potent pro-inflammatory cytokine that is crucial for host defense responses to infection and injury^9^. IL-1β is located in 70–110 kb region of chromosome 2q13–21, including 7 exons and 6 introns. The three most important single nucleotide polymorphisms (SNP) out of at least 20 SNPs that have been reported in the region of IL-1β are −511 (rs16944), + 3954 (rs1143634) and – 31 loci. They exhibit strong influence on gene transcription and the eventual functional alterations^10^. There are several reports on the involvement of cytokines in the pathophysiology of SCD; many of them showing significantly elevated levels of plasma IL-1β in these patients^11^^-^^14^. It is believed that release of cytokines in response to infection, endothelial cell activation and other injurious agents may play key role in the pathophysiology of VOC in SCD^7^.

−511 and +3954 cytokine genes of IL-1β have been postulated to be involved in vaso-occlusion phenomenon of SCD through the processes of inflammation, cellular adhesion, signaling, transport or coagulation^15^. Since studies on the association of cytokines’ polymorphisms with SCD phenotypes are scanty, the present study aimed to estimate the association of IL-1B+3954 polymorphism with the incidence, clinical and laboratory profiles of Egyptian patients with SCD; and to determine whether IL-1B+3954 polymorphism contributes to the clinical evolution of SCD; and to estimate the value of IL-1B+3954 polymorphism as a genetic predictor of SCD clinical heterogeneity.

## MATERIALS AND METHODS

 This cross-sectional study included 50 Egyptian SCD patients (mainly children & adolescents) and 50 age and sex matched healthy control subjects from the same ethnic background. The SCD patients were following in the hematology outpatient clinic at Cairo University Children Hospital (CUCH) during the study period for routine outpatient management. Diagnosis of sickle cell disease was based on hemoglobin electrophoresis and high performance liquid chromatography (HPLC Bio Rad, USA); used to measure several hemoglobin species (Hb F, HbA1, Hb A2 and Hb S).

All patients recruited for the study were in steady state i.e. they had no acute event for at least four consecutive weeks after the last crisis. Those who had been transfused in the three months prior to the study period, and those who had other chronic or inflammatory conditions were excluded. Approval for sampling and participation in the study were taken from all guardians. The study protocol was approved according to the local hospital research guidelines. All procedures were in accordance with the ethical standards of the responsible committee on human experimentation (institutional and national) and with the Helsinki Declaration of 1975, as revised in 2008. The local Medical Ethics Committee of each of the Clinical Pathology Department and the Pediatrics Department, Cairo University approved the study.

Clinical as well as demographic data were obtained from medical records and interviews with the patients. Proper history taking with special attention to age at diagnosis and frequency of blood transfusion was taken. VOC was defined as pain in the extremities, back or abdomen without any other explanation^16^. Occurrence of VOC necessitating hospitalization in the preceding year was considered positive for VOC. Occurrence of osteonecrosis or cholelithiasis at any time of the patient’s life was considered positive. CBC results; as well as results of latest (AST, ALT, Creatinine, Ferritin and Hemoglobin electrophoresis), were documented.

Two ml of whole blood (on EDTA containers) were collected from patients and controls for detection of the IL-1B +3954 polymorphism using polymerase chain reaction and restriction fragment length polymorphism (PCR/RFLP) technique. Genomic DNA was extracted from peripheral blood lymphocytes by ready-made Isolation kit AxyPrep Blood Genomic DNA Miniprep Kit (catalogue number AP-MN-BL-GDA-50; Axygen Biosciences Inc., Ocean City, California, USA). 

Polymerase chain reaction (PCR) was carried out with the forward primer CTC AGG TGT CCT CGA AGA AAT CAA A and Reverse primer GCT TTT TTG CTG TGA GTC CCG. 

**Figure 1:**
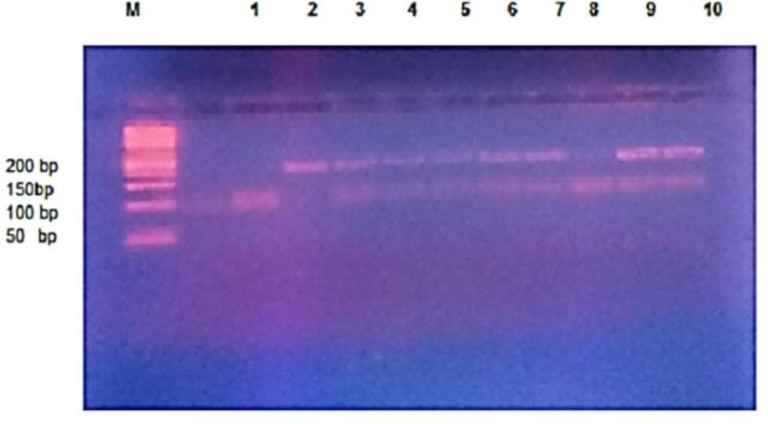
Agarose gel electrophoresis of IL-1 B genotypes: showing DNA marker ladder (M), Lanes1, 8: Wild homozygous CC genotype; lanes 3, 4, 5, 6, 7, 9, 10: Heterozygous C/T Genotype. Lane 2: Homozygous mutant T/T genotype

The optimized PCR conditions included 2 minutes of denaturation at 95 °C followed by 30 cycles of denaturation at 95°C for 30 sec; annealing at 54°C for 30 sec and extension at 72°C for 30 sec. The PCR was completed with a final extension step of 5 min at 72°C. PCR product of 194 base-pair (bp) in size matched on 1.5% agarose gel was subjected to 10 U of *Taq *I restriction enzyme (Promega) at 65°C overnight. The restriction pattern was observed on a 3% agarose gel electrophoresis under UV light after staining with ethidium bromide. The enzyme cut a constant band of 12 bp (the absence of which indicates incomplete digestion) followed by two more bands of 85 bp and 97 bp diagnostic for allele-C, whereas for allele-T, a 12 bp and 182 bp were visualized (Figure1). 

Doppler echocardiography was performed for cases to measure pulmonary artery pressure. Gladwin et al defined pulmonary hypertension as a tricuspid regurgitation jet velocity of at least 2.5 meter per second^17^. Right ventricular systolic pressure (RVSP) could be estimated by measuring the tricuspid regurgitation (TR) jet maximum velocity by spectral Doppler. The right atrial (RA) pressure could be estimated by the respiratory collapsibility of the inferior vena cava. If there was no significant stenosis at the right ventricular outflow tract, or the pulmonary valve, the RVSP was equivalent to the pulmonary artery systolic pressure^18^.

The results of measuring end systolic pulmonary artery pressure (ESPAP) were analyzed into mean, minimum, maximum, and median and comparison of mean ESPAP between different genotypes was performed.

**Statistical analysis**

Data were statistically described in terms of mean ± standard deviation (±SD), median and range, or frequencies (number of cases) and percentages when appropriate.

Comparison of numerical variables between the study groups was done using Student’s t-test for independent samples in comparing two groups when normally distributed and Mann–Whitney U test for independent samples when not normally distributed. For comparing categorical data, x^2^ test was performed. Exact test was used instead when the expected frequency is less than 5. Odds ratio (OR) with its 95% confidence interval (95%CI) was calculated to the different genotypes as well as alleles between the different study groups. P values less than 0.05 were considered statistically significant. All statistical calculations were done using computer program SPSS (Statistical Package for the Social Science; SPSS Inc., Chicago, Illinois, USA) release 15 for Microsoft Windows (2006).

## Results

 The clinical and laboratory characteristics of the studied patients are shown in table 1. The patients’ mean age was 12.24 ± 5.5 years. The ages ranged from 3 years to 26 years with a median of 12 years. Twenty-five cases (50.0%) were males and 25 (50.0%) were females. Regarding the control group, the mean age was 9.38 ± 2.24; 26 (52%) were males and 24 (48%) were females.

**Table 1:** Demographic, Clinical and Laboratory data of the study group

	**Mean ± SD**
Weight (kg)	34.9 ± 15.4
Height (cm)	134.5 ± 28.9
Age at onset (years)	2.6 ± 1.4
Age at first blood transfusion (years)	3.3 ± 2.3
Number of transfusions	12.0 ± 8.2
CBC HB (g/dl) PLT (x 103/cmm) TLC (x 103/cmm) HCT (%) MCV (fl) MCH (pg)	8.75 ± 0.78 355.5 ± 135.6 9.13 ± 2.29 23.26 ± 2.1 98.18 ± 13.8 26.37 ± 4.19
RETICS (%)	6.19 ± 2.78
Ferritin (ng/ml)	381.4 ± 230.2
Haemoglobin electrophoresis HbA% HbF % HbS % HbA 2%	13.39 ± 15.86 16 ± 11.9 68.54 ± 14.09 3.30 ± 4.21
ALT (U/l)	26.52 ± 14.97
AST (U/l)	30.40 ± 14.87
Creatinine (mg/dl)	1 ± 0.4

Doppler echocardiography results showed that ESPAP ranged from 20 to 50 mm Hg, with a mean of 35.80±7.489 mm Hg and a median of 35 mm Hg.

IL-1β +3954 polymorphism was successfully genotyped in all SCD patients and control groups and revealed the following distribution: CC genotype was found in 20 (40%) cases *vs.* 18 (36%) in control group; the CT genotype was found in 17 (34%) cases *vs.* 29 (58%) in control group and the homozygous mutant TT genotype was detected in 13 (26%) cases *vs.* 3 (6%) in control group. This genotype distribution yielded a statistically significant difference between both groups (p=0.009) (Figure1), (Table 2).

However, no significant difference of C/T allele distribution was detected between the two groups [p value: 0.246, OR was 1.4, (0.792 – 2.479)]. Moreover, there was no significant difference between the frequency of occurrence of CC genotype and the frequency of the combined genotypes TT and CT in cases and controls **[**(p value= 0.680), OR (95% CI) 0.844 (0.376 – 1.894)**]** (Table 2).

Contrastingly, a highly significant difference in the frequency of occurrence of TT genotype versus the combined genotypes CC and CT in cases and controls was found [(p value= 0.006), OR (95% CI): 5.505 (1.460-20.756)**]** i.e. the homozygous mutant genotype TT was more prevalent in cases compared to control group (Table 2).

**Table 2:** IL-1β +3954 SNP Genotype and Allele Frequencies in Patients and Control groups

**IL-1β +3954 SNP **	**Cases n (%) (n=50)**	**Control n (%) (n=50)**	**p-value**	**OR (95% CI)**
**CC (wild)**	20 (40%)	18 (36%)	*0.009	
**CT (heterozygote)**	17 (34%)	29 (58%)		
**TT (mutant)**	13 (26%)	3 (6%)		
**C/T**	57 (57%)/43 (43%)	65 (65%)/35 (35%)	0.246	1.4 (0.792– 2.479).
**CC vs. TT+CT**	20(40%)/30(48.4%)	18(36%)/32 (64%)	0.680	0.844(0.376- 1.894)
**TT vs. CC+CT**	13(26%) /37 (74%)	3(6%) /47 (94%)	*0.006	5.505(1.460-20.756)

* p<0.05 is considered statistically significant.

No association was found between IL-1β +3954 genotypes and age (p = 0.087), age at diagnosis (P =0.125), age at first blood transfusion (p =0.318), number of transfusions (P = 0.572), weight (P =0.228) or height (P=0.445) (Table 3).

**Table 3:** Association between IL-1β +3954 Genotypes and the Demographic Data

	** IL-1β +3954 genotypes**			**P-value**
	**CC (n=20)**	**CT (n=17)**	**TT (n=13)**	
Age (mean ± SD) (years)	14.05 (± 5.1)	10.12 (± 5.9)	12.23 (± 5.0)	0.087
Age at onset (mean ± SD) (years)	2.53(±0.835)	2.06(±0.65)	3.69 (± 2.39)	0.125
Age at 1st blood transfusion (mean ± SD) (years)	3.28 (± 1.16)	3.00 (± 2.47)	4.00 (±3.53)	0.318
Number of transfusions (mean ± SD) (times)	13.05 (± 5.68)	11.12 (± 9.77)	11.62 (± 9.63)	0.572
Weight (mean ± SD) (kg)	38.45 (± 16.05)	29.62 (± 13.73)	36.54 (±15.70)	0.228
Height (mean ± SD) (cm)	139.64 (± 22.24)	125.53 (± 34.34)	137.23 (± 30.150)	0.445

*p<0.05 is considered statistically significant.

No significant association was found between IL-1β +3954 genotypes distribution and VOC (p= 0.321), cholelithiasis (p= 0.557), or osteonecrosis (Table 4). 

**Table 4:** Cholelithiasis, VOC and Osteonecrosis in SCD patients with different IL-1β +3954 Polymorphism Genotypes

	**Genotype**	**P-value**	**OR (95% CI)**
VOC (n=49)	CC 19 (38.8%)	0.465	
	CT 17 (34.7%)		
	TT 13 (26.5%)		
	CC#TT+CT	0.216	4.69 (0.18-121.0)
	TT#CC+CT	0.358	1.12 (0.04-28.93)
Osteonecrosis (n=4)	CC 1 (25%)	0.557	
	CT 2 (50%)		
	TT 1 (25%)		
	CC#TT+CT	0.407	2.11 (0.2-21.87)
	TT#CC+CT	0.747	0.9 (0.09-9.97)
Cholelithiasis (n=4)	CC 2 (50%)	0.321	
	CT 1 (25%)		
	TT 1 (25%)		
	CC#TT+CT	0.670	0.642 (0.08-4.98)
	TT#CC+CT	0.962	0.9 (0.09-9.97)

*p<0.05 is considered statistically significant.

Comparison of the mean ESPAP among the different genotypes showed statistically significantly higher mean ESPAP among TT versus the combined genotypes CC + CT (TT#CT+CC) (42.62 # 33.49) (P value: <0.001) (Table 5).

Comparison of laboratory values among the different genotypes did not show statistically significant difference (Table 6).

**Table 5:** Mean ESPAP in SCD patients with different IL-1β +3954 Polymorphism Genotypes

	**Mean ESPAP (mmHg)**	**P-value**
CT+TT CC	37.72 33.9	0.542
TT CC+CT	42.62 33.49	<0.001*

* p<0.05 is considered statistically significant

**Table 6:** Associations between IL-1β +3954 Polymorphism Genotypes and Laboratory Values

	**IL-1β +3954 polymorphism genotypes**			**P-value**
	CC (n=20)	CT (n=17)	TT (n=13)	
HB (mean ± SD) g/dl	8.68 (± 0.725)	8.72 (± 0.84)	8.90 (± 0.84)	0.765
RETICS (mean ± SD) %	5.69 (± 2.45)	6.09 (± 2.73)	7.09 (± 3.28)	0.431
HCT (mean ± SD) %	23.03(± 1.08)	23.7 (± 2.4)	23.3 (± 2.3)	0.528
MCV (mean ± SD) fl	82.42 (± 11)	79 (± 11)	147 (± 22.1)	0.219
MCH (mean ± SD) pg	26.2 (± 3.7)	25.17 (± 4.2)	28.12 (± 4.5)	0.414
TLC (mean ± SD) x 103/cmm	8.7(± 2)	9.04 (± 2.70)	9.87 (± 2.08)	0.189
PLT (mean ± SD) x 103/cmm	391.3 (± 150)	325.4 (± 11)	339.8 (± 13)	0.45
Ferritin (mean ± SD) ng/ml	372.7 (± 149.7)	404.4 (± 301)	359.6 (± 241.6)	0.760
HbS (mean ± SD) %	65.64 (± 17.9)	71.06 (± 11)	69.70 (± 9.5)	0.644
HbF (mean ± SD) %	19.1 (± 14.6)	11.8 (± 9.5)	16.8 (± 9.2)	0.221
HbA1 (mean ± SD) %	14.1 (± 19)	14.3 (± 15.2)	10.8 (± 11)	0.896
HbA2 (mean ± SD) %	2.6 (± 0.6)	4.4 (± 7.1)	2.8 (± 0.6)	0.65

*p<0.05 is considered statistically significant.

## Discussion

 SCD is one of the most common severe monogenic disorders worldwide. The underlying molecular defect is a single nucleotide substitution (βS– HBB; GAG>GTG; glu → val; rs334) in the gene that encodes the β globin chain of hemoglobin. The resulting hemoglobin S (Hb S) polymerizes when deoxygenated, causing polymer-associated lesions of the red blood cells. The World Health Organization recognizes SCD as a global public health problem, as the overall number of babies born with SCD between 2010 and 2050 is estimated to be about 14.24 million^19^.

Interleukin-1β (IL-1 β) is a potent pro-inflammatory cytokine that is crucial for host defense responses to infection and injury. IL1 gene variations have been shown to influence the risk of disease progression in many chronic illnesses like SCD, rheumatoid arthritis, inflammatory bowel disease, cardiovascular disease, osteoporosis and periodontitis**.** The increase in IL-1β cytokine may lead to increase in inflammation. Variations in the upstream region of the IL1B gene have been associated with several diseases and IL1β protein expression^20^. IL-1β-mediated diseases are often called “auto-inflammatory” and the dominant finding is the release of the active form of IL-1β driven by endogenous molecules acting on the monocyte/macrophage^21^.

To our knowledge, this is the first study to address this type of polymorphism in Egyptian SCD patients. However, an earlier study was conducted among Brazilian patients and their findings were different from ours^14^.

Our study revealed that the homozygous mutant TT genotype was significantly higher in cases compared to control group (P-value= 0.009) (Table 4). Moreover, the frequency of occurrence of the homozygous mutant TT genotype versus the combined CC and CT genotypes was significantly higher among cases than controls *(p* value: 0.006) (Table 4). These results are different from the results of the study done by Vicari et al., 2015 who reported that patients with SCD and the control group had similar IL-1β +3954 genotype distribution^14^**.**

However, our study did not show statistically significant difference in allelic distribution between cases and controls (p=0.246) (Table 4) and this is similar to the finding of Vicari and his colleagues.^14^

The current study showed no association between IL-1β +3954 genotypes and different demographic data as: age; gender; age at diagnosis; height, or weight (Table 4). We did not find a statistically significant difference between IL-1β +3954 genotypes and VOC, osteonecrosis, cholelithiasis or laboratory characteristics (Tables 4 & 6). However, a significant association was observed between IL-1β +3954 SNP and mean ESPAP. The mean ESPAP was significantly higher among TT genotype versus the combined genotypes CC + CT (Table 5). This finding implies that SCD patients with homozygous mutant TT genotype should be more closely monitored for pulmonary hypertension. This confirms the observation of Vicari and colleagues who similarly reported that IL-1β +3954C > T SNP was associated with increased risk of elevated pulmonary arterial pressure among SCD Brazilian patients. Interestingly, Vicari and colleagues reported increased risk of osteonecrosis among SCD patients carrying IL-1β +3954C > T SNP. This association was discordant with our results. This discordance could be owed to the small number of patients having this complication in our SCD population^14^.

## CONCLUSION

 The present study showed that there is an increased prevalence of the mutant genotype of IL-1β +3954 SNP in Egyptian SCD patients. Regarding disease complications, the mutant genotype was more prevalent in cases complicated by pulmonary hypertension. These findings point to the possible role of IL-1β +3954 SNP in the pathophysiology of the disease and its manifestations. Expansion of this study on a larger number of patients is recommended for further reinforcement of our results; as well as study of other genes related to disease susceptibility and prognosis in the Egyptian population. Further testing is required to determine the validity of the shown association between the mutant IL-1β genotypes TT, CT and the occurrence of other complications (leg ulcer, priapism, acute chest syndrome, splenic sequestration, retinopathy and stroke) in SCD patients. 
